# A Rare Case of the Newly Recognized Kaposi Sarcoma Herpesvirus-Associated Disease

**DOI:** 10.7759/cureus.8784

**Published:** 2020-06-23

**Authors:** Qunfang Li, Faria Ali, Vivek Kak, Richard Santos

**Affiliations:** 1 Hematology and Oncology, Henry Ford Health System, Detroit, USA; 2 Internal Medicine, Henry Ford Allegiance Health, Jackson, USA; 3 Infectious Disease, Henry Ford Allegiance Health, Jackson, USA

**Keywords:** hiv, kshv inflammatory cytokine syndrome (kics), multicentric castleman disease (mcd), interleukin (il)-6, il-10

## Abstract

Kaposi sarcoma herpesvirus (KSHV) is associated with Kaposi sarcoma (KS), primary effusion lymphoma, and multicentric Castleman disease (KSHV-MCD) in patients infected with human immunodeficiency virus (HIV). We present a case consistent with a newly recognized KSHV inflammatory cytokine syndrome (KICS), distinct from KSHV-MCD. Although both disorders exhibit signs of substantial inflammation, KICS has minimal lymphadenopathy/splenomegaly and negative pathologic nodal changes in the setting of low CD4 count. KICS is easily misdiagnosed as severe sepsis or other KS-related diseases in HIV/AIDS patients and carries a high mortality. ​Standard therapy is still under investigation due to its rarity, whereas the treatment regimen for KSHV-MCD may lead to clinical remission. Early recognition and prompt management are crucial to improve the survival of the under-recognized KICS.

## Introduction

Kaposi sarcoma herpesvirus (KSHV), a human gamma herpesvirus 8 (HHV8), is associated with Kaposi sarcoma (KS), primary effusion lymphoma (PEL), hemophagocytic lymphohistiocytosis (HLH), and multicentric Castleman disease (KSHV-MCD) in patients infected with human immunodeficiency virus (HIV) [1-3]. As a rare and unique entity, KSHV inflammatory cytokine syndrome (KICS) was first recognized in patients infected with HIV in 2010 and is distinct from KSHV-MCD [2,4,5]. Uldrick et al. initially described six patients with a co-infection of HIV and HHV8, who presented with classic KSHV-MCD signs and symptoms, however, without pathologic evidence or radiographic findings associated with MCD [4,6]. All of those patients had features of leak syndrome, resulting in pulmonary infiltrates, respiratory distress, and anasarca, along with significant systemic inflammatory responses as the hallmark of this syndrome [1].

The lytic phase of HHV8 is characterized by massive stimulation and production of viral proteins, including viral IL-6 (vIL-6). As a homolog of human IL-6 (hIL-6), vIL-6 has similar biologic functions and structural characteristics. Although less potent, vIL-6 recruits macrophages and neutrophils, suppresses regulatory T-cell function, and induces tissue damage and hepatic acute phase response, by binding to the ubiquitous gp-130 transmembrane protein in human cells. Thus, it can still exert its effects despite bypassing the actual IL-6 receptor [5]. Moreover, vIL-6 also stimulates further production of hIL-6 in uninfected cells [7]. In HIV-related MCD, the hIL-6 and vIL-6 expression can initiate uncontrolled cytokine cascade in the host, and induce a disproportionate inflammatory response [1,2]. KSHV-associated miRNAs also induce IL-10 production [8]. Production of vIL-6 and induction of hIL-6 both contribute to KICS symptoms, perhaps in a combination of overproduction of IL-10 and other cytokines [5].

Because of its rarity, KICS is easily misdiagnosed as severe sepsis or other KS-related disorders in HIV/AIDS patients and is, therefore, probably under-recognized [1]. KICS symptoms are nonspecific and almost identical to KSHV-MCD; in addition, its laboratory abnormalities resemble KSHV-MCD by hypoalbuminemia, hyponatremia, cytopenia, increased C-reactive protein, high KSHV viral load (VL), and elevated IL-6 and IL-10 [4]. KICS also mimics severe sepsis and acute respiratory distress syndrome (ARDS), often requiring ventilator and vasopressor support, but antibiotics do not help. Overall, recent case reports demonstrate a high mortality rate (about 60%) in patients diagnosed with KICS [1,4,5]. Awareness and a high index of suspicion in patients with pre-existing HIV and KS will lead to earlier diagnosis and prompt treatment [1,4,5,9]. Here we describe a case consistent with KICS, where the patient, unfortunately, succumbed to complications of multiorgan involvement in one month.

## Case presentation

**Figure 1:**
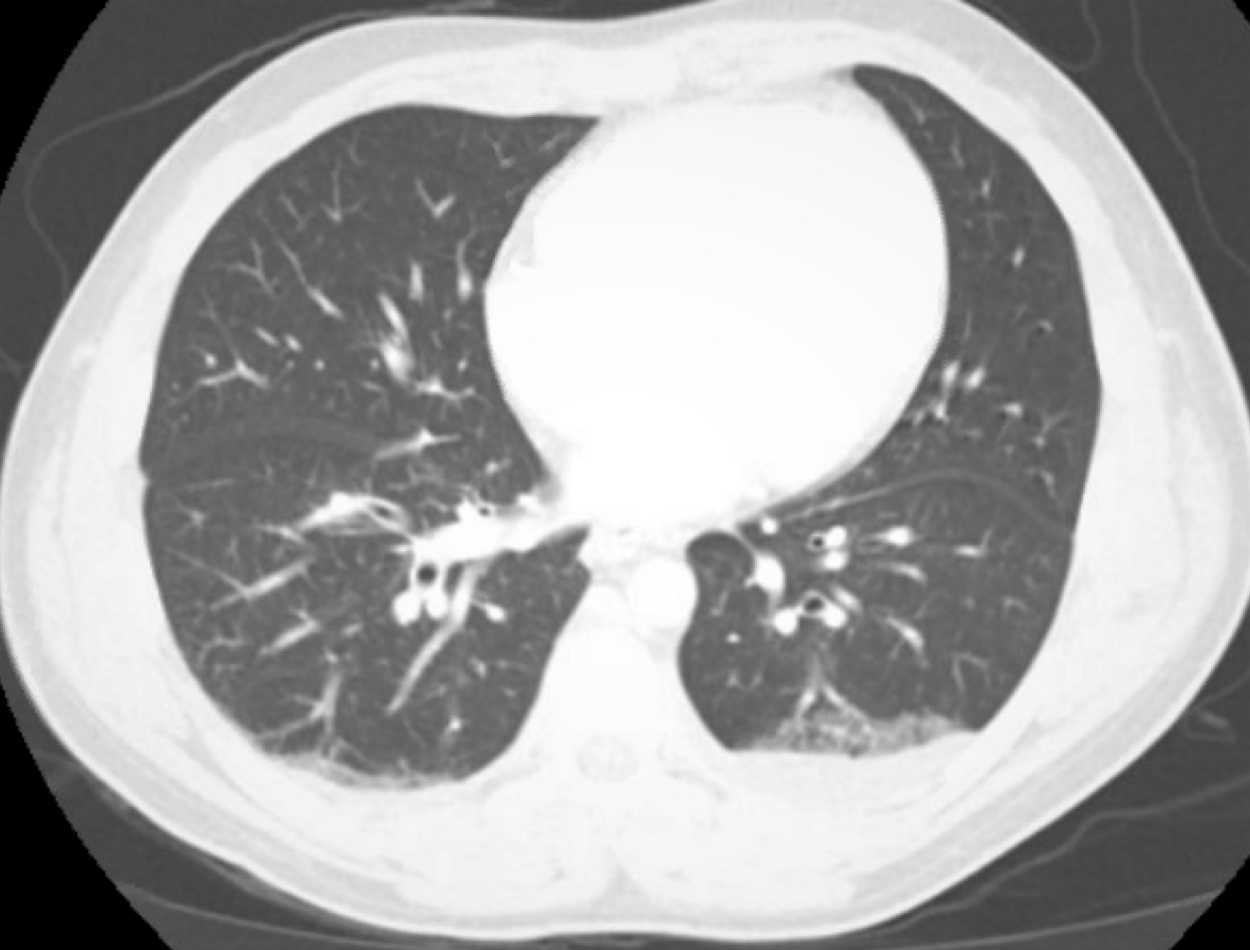
CT of the chest shows small bilateral pleural effusions and bibasilar atelectasis with no lymphadenopathy.

**Figure 2:**
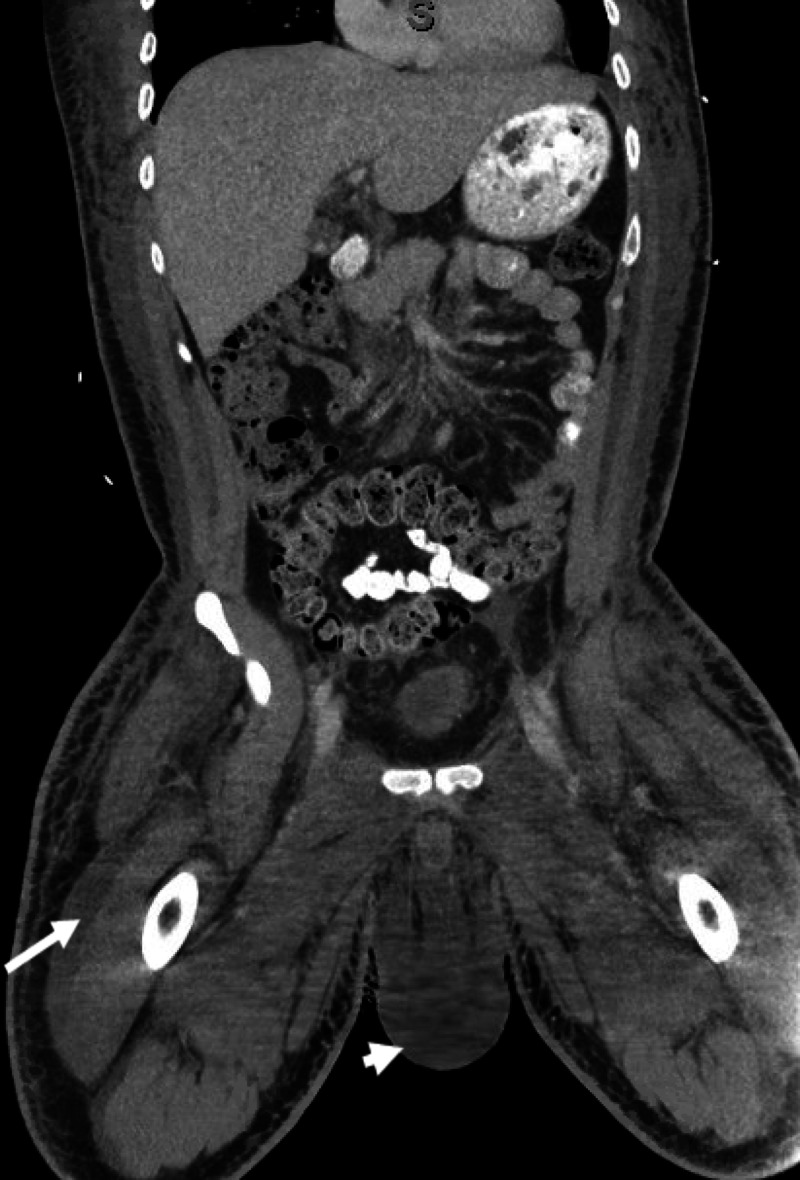
CT of the abdomen and pelvis shows no enlarged lymph node. There is severe generalized soft tissue anasarca in scrotal wall (white arrow head) and further extended into the visualized proximal legs (white arrow).

A 33-year-old African American male with a prior medical history of syphilis, HIV/AIDS on antiretroviral therapy (ART), and stage IV KS on doxorubicin was admitted due to worsening fatigue, bilateral leg swelling, and hyponatremia. He also had multiple complaints, including headaches, severe muscle and body aches, abdominal pain, nausea, vomiting, altered bowel habits, and shortness of breath. His chronic lower extremity edema had been getting worse with extension to his scrotum, he gained 20 pounds in two weeks before admission, and was unable to ambulate. His HIV/AIDS had been treated with Triumeq for more than one year, and his HIV VL was less than 20 copies/ml. However, his CD4 count was still low at 58 cell/µl not long before admission. Last doxorubicin for his KS was two months before. He did have mild lymphadenopathy at that time, while a left supraclavicular lymph node biopsy did not show any lymphoproliferative process or coexistent MCD. A repeat CT scan of his chest, abdomen, and pelvis upon current admission did not show any enlarged lymph nodes or hepatosplenomegaly except small bilateral pleural effusions and anasarca (Figures 1, 2).

**Figure 3:**
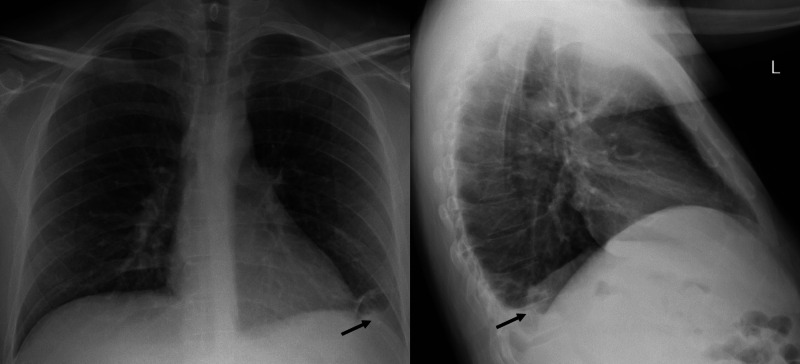
Chest x-ray upon admission shows a small left basilar opacity (black arrow).

Upon presentation, he was febrile at 102.6°F and chronically ill-appearing with dry mucous membranes. He had a distended abdomen as well as significant scrotal swelling in addition to 2+ pitting edema on his bilateral lower extremities. A small condyloma was noticed near his gluteal fold. Hyperpigmented lesions consistent with KS were noted all over his body, more significant on the lower extremities. His initial pertinent labs showed a white blood cell (WBC) count of 6 K/µl, sodium 122 mmol/L, albumin 1.8 g/dl, and lactic acid 1.7 mmol/L. Additional workups demonstrated negative urinalysis and no pneumonia on chest x-ray (CXR), although a left basilar opacity was consistent with a small pleural effusion (Figure 3). Abdominal CT indicated evidence of peripancreatic edema and minimal edema in the sigmoid colon. Pancreatitis was later ruled out by a normal lipase. The patient was placed on a sepsis protocol with IV fluid and started on ceftriaxone and metronidazole for assumed intra-abdominal infection. He was also given diuretics and albumin infusion for his anasarca. The patient continued to be febrile although his antibiotics regimen was escalated to vancomycin and piperacillin/tazobactam later, whereas his blood and scrotal sac drainage culture had remained negative. Furthermore, no infectious source was established after extensive negative workups for bacteria, fungal, and other viral infections.

The patient's HIV treatment with Triumeq and antibiotic prophylaxis with azithromycin and trimethoprim-sulfamethoxazole were continued. While initially at baseline, his liver enzymes started trending upwards and he continued to have GI symptoms. A colonoscopy with biopsy of his rectal lesion was performed, which showed condyloma acuminatum with low-grade and focal intermediate grade anal intraepithelial neoplasia and concurrent ulcer with herpes virocytes. His HIV profiles were repeated, and his CD4 count remained low at 33 cell/µl with mildly increased HIV-1 viral load at 222 copies/ml (Table 1). His Triumeq was changed to Genvoya due to concerns of refractory HIV infection and GI side effects of Triumeq.

**Table 1:** Laboratory abnormalities of Kaposi sarcoma inflammatory cytokine syndrome (KICS) in our HIV/HHV8 co-infected patient. HHV8, human gamma herpesvirus 8.

		Reference range
HIV-1	222	<20 copies/ml
CD4	33	443-1,471 cell/μl
CD4%	5%	35%-66%
CD8	357	190-832 cell/μl
CD8 %	58%	9-37%
CD4/CD8 ratio	<0.1	1.0-3.7
Hemoglobin	5.7	13.5-17.0 g/dl
Platelet	47	150-450 k/μl
Sodium	121	135-145 mmol/L
Albumin	1.65	3.2-4.6 g/dl
C-reactive protein	17.9	<0.5 mg/dl
Interleukin-6	52	≤5 pg/ml
Interleukin-10	2,600	≤18 pg/ml
HHV8	14,855	<1,000 copies/ml

**Figure 4:**
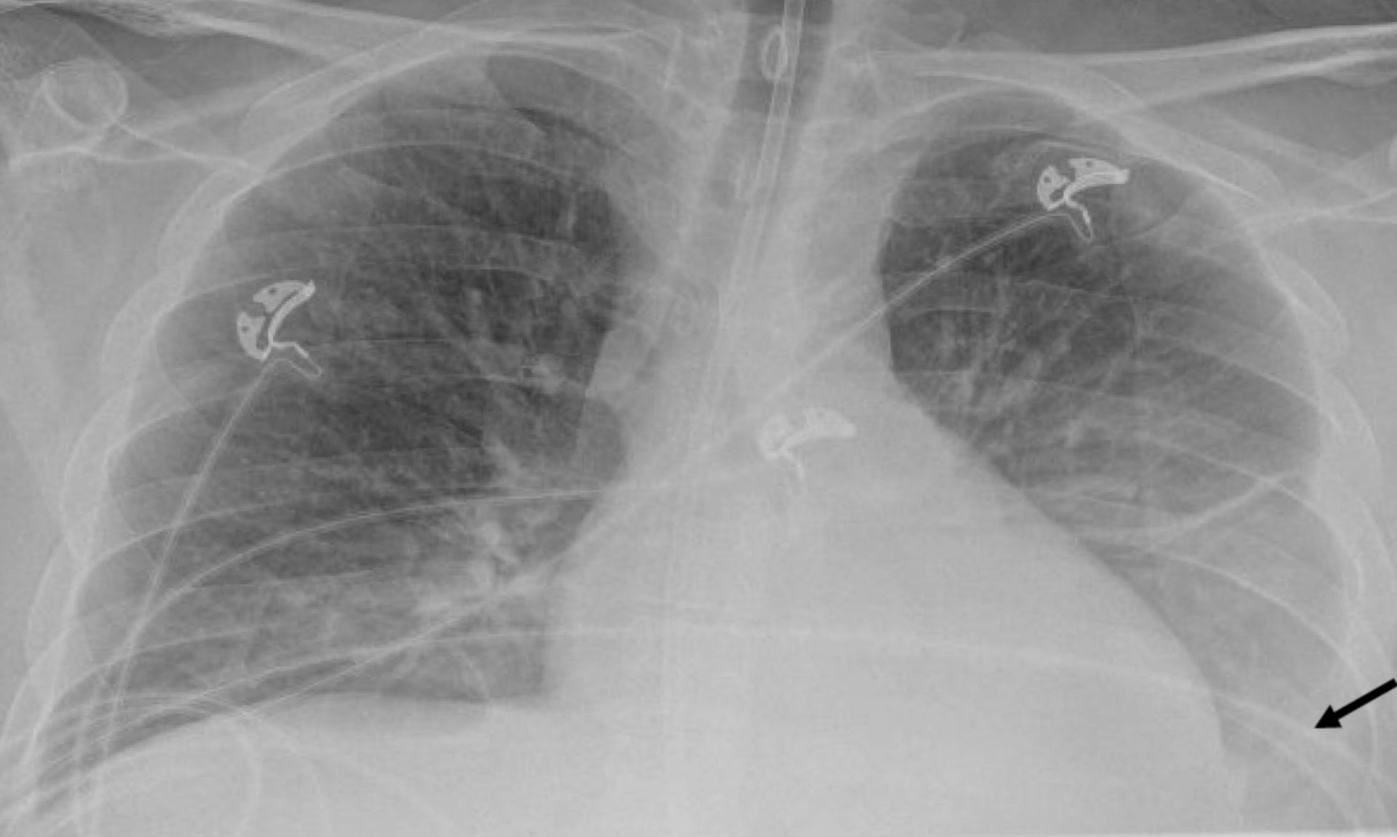
Chest X-ray shows increased haziness of left lower zone due to left lower lobe volume loss and pleural effusion (black arrow).

**Figure 5:**
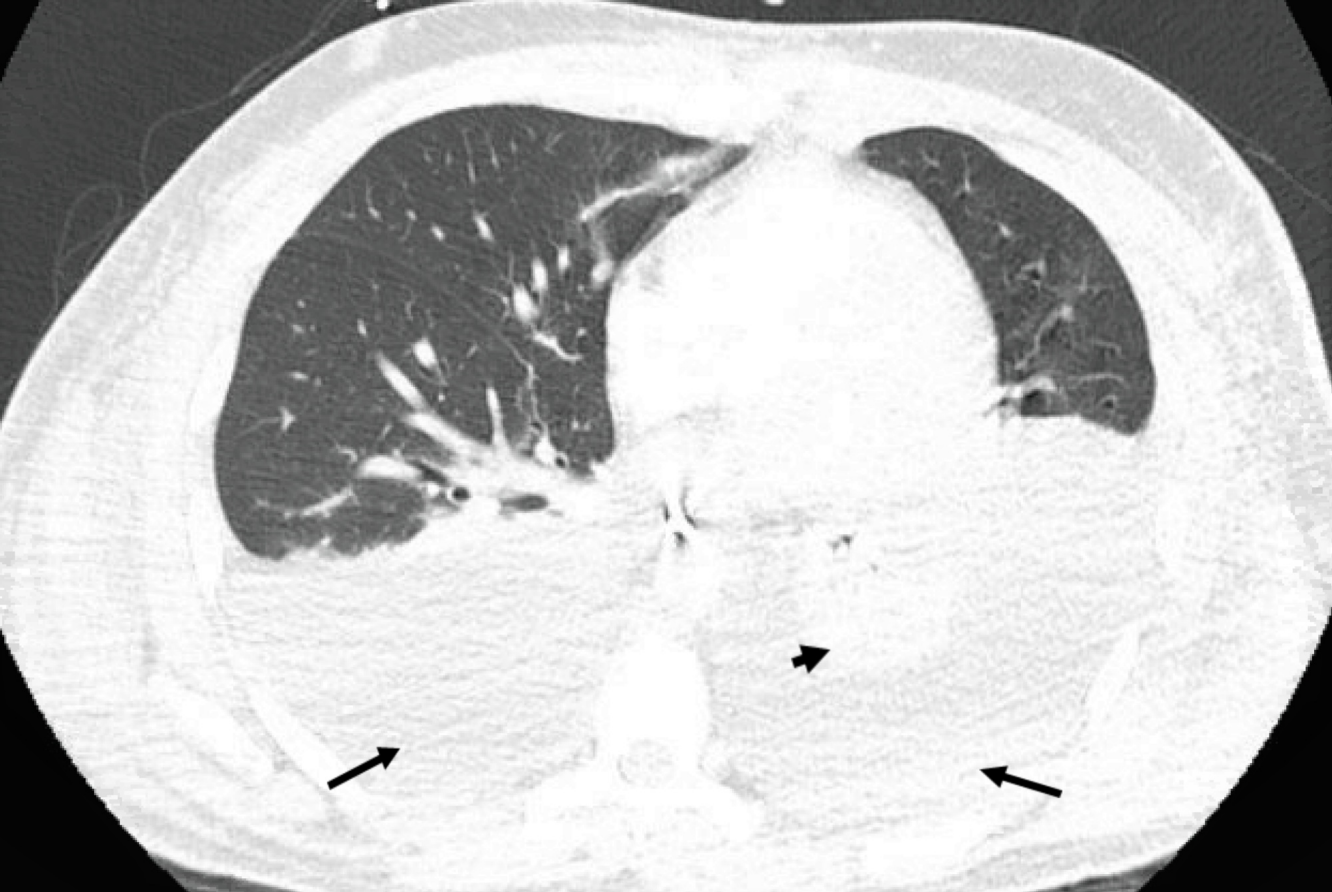
CT of the chest shows large bilateral pleural effusions slightly more on the left side (black arrows) with total consolidation of the left lower lobe (black arrow head). There is no evidence of lymphadenopathy.

The patient had persistent hyponatremia (121-127 mmol/L) with low urine sodium, and did not respond to dose titration of tolvaptan and sodium tablets. His hypoalbuminemia was worsening despite an albumin infusion. His kidney function deteriorated during his hospital course, leading to acute kidney injury with electrolyte abnormalities. In addition, he developed severe anemia with a hemoglobin of 5.7 g/dl and thrombocytopenia of 47,000/μl (Table 1), and then became transfusion dependent. He eventually progressed to acute respiratory failure, and required intubation and medical intensive care unit (MICU) care. His CXR and CT chest demonstrated larger bilateral pleural effusions and left lung consolidation with no lymphadenopathy characteristically seen in MCD (Figures 4, 5). He had therapeutic thoracentesis, and the fluid was negative for malignant cells in his pleural effusion.

His significantly increased C-reactive protein (17.9 mg/dl) and the absence of lymphadenopathy/splenomegaly on CT images led to the suspicion of KICS. KICS was further confirmed by profoundly elevated IL-6 at 52 pg/ml, IL-10 at 2,600 pg/ml, and HHV8 VL at 14,855 copies/ml (Table 1). Despite aggressive immunoglobulin therapy and supportive treatments by our multidisciplinary team, the patient died of multiorgan failure a few weeks later.

## Discussion

We presented a case consistent with the newly recognized KICS in a HIV/HHV8 co-infected patient. The working diagnostic criteria for KICS have been developed by Polizzotto’s group [2,4,5]. KICS is defined with minimal lymphadenopathy/splenomegaly and negative pathologic nodal changes in the setting of lower CD4 count (<100 cells/μl). KICS is unique and distinct from KSHV-MCD although both disorders exhibit substantial inflammatory dysregulation and the same characteristic IL-6/IL-10 signature attributable to KSHV lytic activation [5,10]. Fevers, cytopenia, cachexia, hypoalbuminemia, and hyponatremia are the result of the tremendously increased cytokine production [11]. The IL-6 and IL-10 overexpression synergistically contributes to the unchecked activation of inflammation and the transformation of KSHV-related malignancies [8]. This massive cytokine cascade mediates both KICS and MCD, which are considered separate manifestations of the same disease spectrum.

Diagnosis of KICS can be challenging because of an overlapping symptom complex and findings of high KS viremia similar to KSHV-MCD. Nonetheless, multiple aspects can differentiate KICS from KSHV-MCD. Histopathologic findings in KICS are mainly reactive lymphocyte hyperplasia and scattered KSHV-infected plasma cells in both bone marrow and lymph node biopsy. Both KSHV and HIV viremia are similarly high in KICS [12]. In contrast, KSHV-MCD is defined as a lymphoproliferative disorder characterized by KSHV-infected plasmablasts accompanied by recurrent flares of systemic inflammation and KSHV viremia [13]. Histopathologic findings exhibit KSHV-infected, polyclonal plasmacytoid cells in the interfollicular area and hypocellular germinal centers in a lymph node [12]. KSHV viremia during symptoms remains high, whereas HIV viremia is typically low in KSHV-MCD [12]. Finally, KICS is also distinguished from KSHV-MCD by their CD4 counts, where CD4 cells in KICS are typically much lower (<100 cells/μl) than in KSHV-MCD (>200 cells/μl) [2].

KICS is uncommon with less than 20 cases confirmed so far. It can be easily misdiagnosed in immunocompromised patients with HIV/AIDS; differential diagnoses include septic shock, KSHV-MCD, PEL, and KS-related immune reconstitution inflammatory syndrome (KS-IRIS) [2,9]. Comprehensive workups failed to find any other infection source in our patient. His multiple imaging studies revealed no lymphadenopathy and an earlier lymph node biopsy had no classic histopathologic evidence of MCD, thus not supporting a diagnosis of KSHV-MCD. As another KSHV-associated manifestation, PEL is seen in approximately 4% of HIV-associated non-Hodgkin’s lymphoma. PEL generally presents in a liquid phase in body cavities as a lymphomatous growth and may coexist with other KSHV-related diseases. Typically, HIV-positive males with a decreased CD4 cell count such as our patient have a higher risk for PEL development [14]. Although our patient did develop bilateral pleural effusions, PEL was excluded by negative cytology of pleural fluid. On the other hand, KS-IRIS presents as acute deterioration of KS involvement of new systems (gastrointestinal, pulmonary, lymphatic) after starting ART [2,15]. A temporal association within 14 weeks of ART initiation usually exists, while KSHV and HIV viremia remains low accompanied by >50 cells/μl CD4 count increases from pre-ART levels [2,16]. KS-IRIS is unlikely in this patient as he had been on ART for more than one year with substantial KSHV viremia in the setting of very low CD4 cell counts during his entire hospitalization.

The prognosis of KICS is poor compared to KSHV-MCD with a high mortality rate at 60%, is often refractory to available treatment, and carries a greater relapse rate after a short remission period [2,5]. The median overall survival from the time of diagnosis according to a prospective study of 10 patients conducted by Polizzotto et al. was 13.6 months. Anemia and hypoalbuminemia at presentation were independent risk factors associated with early death [4]. Standard therapy is still under investigation due to its rarity and known aggressive course. No approved management algorithm is available currently [2]. Up-to-date management has been essentially focused on maintaining the balance between tumor control and immunosuppression. Thus far, treatment regimens parallel to those used for KSHV-MCD have been the most logical approach [9]. A combination treatment of the anti-CD20 agent rituximab (targeting the B-cell KSHV reservoir), pegylated liposomal doxorubicin (PLD) (chemotherapy for KS suppression), and valganciclovir (antiviral) has been shown to lead to temporary clinical remission [9]. Derivatives of high-dose valganciclovir and zidovudine are cytotoxic compounds that kill KSHV-infected plasmablasts; therapies with these two drugs also demonstrated transient clinical response [17]. Pomalidomide, a thalidomide derivative, is being used as an anti-angiogenic immunomodulator in clinical trials in combination with doxorubicin since single agents alone are not usually sufficient to treat KICS [3]. Other new treatment modalities, including sirolimus (MTOR inhibitor) and tocilizumab (recombinant anti-human IL-6 receptor monoclonal antibody), may represent possible future alternatives [3,18]. Tocilizumab specifically blocks hIL-6 autocrine and paracrine overproduction, therefore inhibiting KS cell proliferation in response to IL-6 stimulation and may be promising for KICS treatment [3,18]. Nonetheless, in HIV/KS co-infected patients with fever of unknown origin, physicians must include KICS in their differential diagnosis to ensure appropriate diagnosis and prompt treatment initiation.

## Conclusions

As a rare and distinct syndrome, KICS can often be confused with severe sepsis and carries a high mortality. Interestingly, unlike other opportunistic infections, it can persist despite good control of HIV viremia with ART. ​Early identification and immediate treatment initiation are crucial to improve survival of this under-recognized KSHV-associated disease. It is therefore imperative that physicians are aware and familiar with KICS, so that the diagnosis of KICS will not be missed or delayed when patients present with systemic signs of inflammation and have concurrent advanced HIV/AIDS along with KS. Moreover, current ongoing clinical trials and research may shed more light on this newly recognized entity by targeting the overproduction of vIL-6/IL-10 and dysregulation of systemic inflammation.
